# Comprehensive Analysis of TCR-β Repertoire in Patients with Neurological Immune-mediated Disorders

**DOI:** 10.1038/s41598-018-36274-7

**Published:** 2019-01-23

**Authors:** Alessandra de Paula Alves Sousa, Kory R. Johnson, Joan Ohayon, Jun Zhu, Paolo A. Muraro, Steven Jacobson

**Affiliations:** 1 Neuroimmunology Branch, Viral Immunology Section, National Institute of Neurological Disorder and Stroke, NIH, Bethesda, Maryland United States; 2 Bioinformatics Section, National Institute of Neurological Disorder and Stroke, NIH, Bethesda, Maryland United States; 3 Clinical Neuroimmunology Unit, National Institute of Neurological Disorder and Stroke, NIH, Bethesda, Maryland United States; 4 Systems Biology Center, National Heart Lung and Blood Institute, NIH, Bethesda, Maryland United States; 5 Division of Brain Sciences, Faculty of Medicine, Imperial College London, London, United Kingdom

## Abstract

In this study we characterized the TCR repertoire profiles in patients with chronic progressive inflammatory neurological disorders including HAM/TSP, associated with human T-cell lymphotropic virus type I (HTLV-I) infection, and multiple sclerosis (MS), an inflammatory, demyelinating disease of the CNS of unknown etiology. We hypothesized that a T-cell receptor (TCR) clonal repertoire ‘signature’ could distinguish HAM/TSP patients from healthy controls, as well as from patients with a more heterogeneous CNS-reactive inflammatory disease such as MS. In this study, we applied an unbiased molecular technique – unique molecular identifier (UMI) library-based strategy to investigate with high accuracy the TCR clonal repertoire by high throughput sequencing (HTS) technology. cDNA-TCR β-chain libraries were sequenced from 2 million peripheral mononuclear cells (PBMCs) in 14 HAM/TSP patients, 34 MS patients and 20 healthy controls (HC). While HAM/TSP patients showed a higher clonal T-cell expansion compared to MS and HC, increase of the TCR clonal expansion was inversely correlated with the diversity of TCR repertoire in all subjects. In addition, longitudinal analysis of TCR repertoires from HAM/TSP patients demonstrated a correlation of the TCR clonal expansion with HTLV-I proviral load. Surprisingly, MS patients showed a higher diversity of TCR repertoires than other groups. Despite higher TCR clonal expansions in HAM/TSP patients, no disease-specific TCRs were shared among patients. Only non-shared or “private” TCR repertoires was observed. While no clones that shared the same CDR3 amino acid sequences were seen in either HC or MS patients, there was a cluster of related CDR3 amino acid sequences observed for 18 out of 34 MS patients when evaluated by phylogenetic tree analysis. This suggests that a TCR-repertoire signature may be identified in a subset of patients with MS.

## Introduction

Human T-cell lymphotropic virus type I (HTLV-I) is the causative agent in a progressive neurologic disease termed HTLV-I-associated myelopathy/tropical spastic paraparesis (HAM/TSP)^1^. It is a chronic inflammatory immune-mediated disease of the central nervous system (CNS), associated with increased HTLV-I proviral loads (PVL) in blood and cerebrospinal fluid (CSF)^2^. Approximately 20–30 million people are infected worldwide. While most remain asymptomatic, a small proportion develop clinical disease including adult T cell lymphoma (ATL), HAM/TSP and other autoimmune inflammatory disorders^3^. In HAM/TSP, circulating HTLV-I antigen-specific T-cells have been shown to cross the blood-brain barrier, infiltrate the spinal cord and are thought to initiate an immunopathogenic response against virus and/or components of the CNS^4^. Similarly, trafficking of T-cells from the peripheral blood into the CNS is also a hallmark of a more common inflammatory, demyelinating disease, multiple sclerosis (MS)^5^. Indeed, the primary progressive form of MS is clinically similar to HAM/TSP^6^ and even, to this day, many patients with HAM/TSP are misdiagnosed as MS. However, unlike HTLV-I mediated HAM/TSP, the antigen(s) driving the inflammatory autoimmune-mediated response in MS patients are still unknown although viruses have long been considered potential environmental ‘triggers’ in this disorder^7^. It is therefore been hypothesized that in MS, environmental triggers (including viruses) in genetically susceptible individuals lead to immunopathogenic T cell responses that contribute to the development of disease^8,9^. A comprehensive characterization of the T-cell receptor repertoire (TCR) in MS patients would therefore be of value to determine if a ‘signature’ of TCRs can be identified, particularly in comparison with a clinically similar disease like HAM/TSP in which immunopathogenic T cells triggered by a known virus have been thought to play a role in disease pathogenesis^10^.

With improved TCR-sequencing technological development, efforts in identifying immune T-cell signatures in blood, CSF and brain lesions of MS patients have been initiated, with the goal to better characterize MS disease processes^11^ and to distinguishing subsets of MS patients^12^. Previously, we have reported that the TCR clonal repertoire in the peripheral blood of MS patients was different than what is observed from the T-cell profile within the CSF. Moreover, there was a higher clonal expansion in both peripheral blood and CSF compartments of MS patients compared to controls^13^. In HAM/TSP patients, there are reports on complementarity determining region 3 (CDR3) TCR repertoire analysis in which no significant differences of expanded T-cell clones in HAM/TSP patients were observed when compared to asymptomatic carriers^14^. However, another study had shown that HAM/TSP patients demonstrated a highly expanded T-cell repertoire compared to non-HTLV-I infected individuals^15^. Clearly, additional studies are required to determine the degree of expansion of TCR clonotypes in patients with chronic, progressive neurologic disease.

Over the past few years, accurately evaluating the TCR clonal expansion using high throughput sequencing (HTS) platforms has been challenging due to technical limitations including over sequencing, sequencing errors inherent to HTS, PCR errors [amplification bias] and reverse transcription artifacts, potentially leading to inaccurate results^16^. To overcome some of these hurdles, here, we have applied an innovative barcoding-library strategy to obtain high fidelity profiling of immune repertoires^8^. We have combined the use of unique molecular identifiers (UMIs) which tags each individual cDNA TCR-transcript with a previously published UMI-based TCR-β data sequence analysis pipeline called MiGEC^17^. The rationale for using this robust unbiased technical approach was to accurately compare the clonal expansion of TCR-β repertoires in circulating PBMCs of patients with neurological immune-mediated diseases including HAM/TSP, MS, and healthy control (HC) individuals. Importantly, we wished to more comprehensively address whether a shared T-cell repertoire was present in HAM/TSP or MS patients in comparison with healthy controls, underlying a potential antigen-driven clonal expansion in these patients.

## Materials and Methods

### Subjects

A total of 68 subjects including 14 HAM/TSP patients, 34 MS patients and 20 HC were included for sequencing of TCR β-chain repertoire by HTS. HAM/TSP patients were part of the clinical protocol for natural history of HTLV-I infection (protocol #98N0047), while MS patients were part of the clinical protocol for evaluation of disease progression by magnetic resonance imaging (protocol #89N0045). All patients were recruited by the Neuroimmunology Clinic Unit at the National Institute of Neurological Disorder and Stroke, National Institutes of Health (NIH), Maryland, USA. Healthy controls were volunteers from the blood bank of the NIH. All subjects gave written informed consent before inclusion in accordance with the Declaration of Helsinki and the study was reviewed and approval by the NIH Institutional Review Board. HTLV-I proviral load (PVL) from HAM/TSP patients (range 6.9–38.0%) was quantified based on the percentage of HTLV-I tax gene expression in PBMCs by using the digital droplet PCR technique (ddPCR)^18^. Of 34 MS patients in this study, 3 (MS-S11, MS-S13 and MS-S20) were diagnosed as secondary progressive MS while 31 patients were relapsing remitting MS^19^. Demographic information including age, sex, ethnicity as well as TCR-repertoire dataset statistics from each subject is presented in the Supplementary Section (Table 1) including total number of HTS sequences, number of TCR sequences with a UMI, number of unique TCR-β clonotypes, percentage of clonal expansion and Shannon diversity. These descriptive measures were also interrogated by correlation analysis.

### TCR β-chain library preparation

Peripheral mononuclear cells (PBMCs) were isolated from the peripheral blood according to the Ficoll-Hypaque density gradient centrifugation method and then cryopreserved in liquid nitrogen until the date of use. cDNA TCR-β libraries with a unique molecular barcode-based strategy was generated from 2 million PBMCs, which was previously kept in RNA*later* (Invitrogen, USA) at temperature of −80 °C until RNA extraction. In addition, TCR libraries from 1 million sorted CD4^+^ and CD8^+^ T-cells were included in our analysis. Total RNA was extracted using the RNeasy kit (Qiagen, USA) according to the manufacture’s instruction. cDNA synthesis was performed using the anchored switch 5′rapid amplification of cDNA ends (5′RACE) PCR-based primer combined with an UMI that consists of 10 random nucleotides sequences (AAGCAGTGGTATCAACGCAGAGTAC**NNNNNNNNNN**UCTTrGrGrGrG)^20^.

Each cDNA in the TCR-library was targeted with a unique sequence of 10 nucleotides during reverse transcription. The human TCR VDJ β-chain region was constructed using a pair of primers (12 μM) specific for the TCR constant region and 5′RACE region. Sequence primers used for TCR-library construction is presented in Supplementary Section (Table 2). TCR-library was amplified using KAPA Real-time Library Amplification kit (KAPA Biosystems, USA) following 4 PCR steps: 1 cycle at 95° for 5 min; 5 cycles at 98° for 15 s, 72° for 1 min; 5 cycles at 98° for 15 s, 70° for 10 s, 72° for 1 min; 20 cycles at 98° for 15 s, 68° for 10 s, 72° for 1 min. Libraries were amplified using a SYBER green-based real time, high fidelity PCR master mix, where each PCR plate contained fluorescent reference standards representing a range of distinct DNA concentrations. The 1^st^ PCR reaction was stopped when the TCR product reached an amplification between standard 2 and standard 3. Then, TCR libraries double-stranded were purified using Ampure magnetic beads (Beckman Coulter, USA) according to the manufacture’s instruction. The amplified TCR product, which is ~650 bp, was recovered using a 2% E-gel SizeSelection electrophoresis system (Invitrogen, USA). During the 1^st^ PCR round, a sample index and adaptor sequences necessary for Illumina paired-end sequencing were introduced. In order to increase the DNA amount of TCR libraries, a 2^nd^ PCR was performed as following 1 cycle at 95° for 5 min; 9 cycles at 98° for 20 s, 54° for 30 s, 68° for 1 min; 7 cycles at 98° for 20 s, 68° for 1 min, 1 cycle at 72° for 1 min, using the Kapa Real-Time Library Amplification kit (Kappa Biosystems, USA). Final PCR product was cleaned by XS beads and the quality of TCR library was analyzed using a Bioanalyzer (Agilent Technology, CA, USA). TCR-library concentration was measured on a Qubit Fluorometric Quantitation System (Invitrogen, USA). TCR-β libraries were re-quantified using Kapa Library Quantification kit according to the manufacturer’s instruction (Kapa Biosystems, USA) and a final pooled cDNA amount of 30pmol was obtained for HTS.

### Sequencing of TCR-β repertoire and bioinformatics analysis

TCR-β clonal repertoire on the peripheral blood of HAM/TSP patients, MS patients and HC were sequenced on the HiSeq. 2500 Illumina system platform. Paired-end sequencing was performed by using customized primers that map to the VDJ β-chain genes encoding the TCR region. For the primers used, only R2, representing the 3′reverse complement read, was barcoded (Supplementary Section, Table 2). The sequencing was performed in a 2 × 150 bp modality by the DNA Sequencing Core Facility at National Heart Lung and Blood Institute, National Institute of Health, Maryland, USA. Each final library pool was spiked in with 25–30% phiX to increase sequence diversity and ultimately, data quality.

As over sequencing and sequencing errors inherent to HTS, PCR and reverse transcription can result in artifacts that may lead to inaccuracies, we applied an innovative barcoding-library strategy to obtain an error-free profiling of immune repertoire^17^. Over 200 million TCR-β sequences were generated per run covering 15–18 subjects (NCBI SRA access number GSE121082). After quality filtering, raw sequence reads were then analyzed using the MiGEC software pipeline (http://milaboratory.com/projects/migec-project/). VDJ genes were aligned to the human TCR-β germline sequences based on ImmunoGenetics database (IMGT) and the complementarity determining regions (CDRs) were extracted to investigate the profile of clonotypes (defined as unique CDR3 amino acid sequences and V-J segments genes). Clonal expansion, diversity of the TCR repertoire and the relatedness of CDR3 amino acid sequences across samples were evaluated using VDJtools (www.vdjtools.readthedocs.io/en/latest/). For estimating the diversity of the TCR repertoire, the Shannon Entropy option was used (ShannonWienerIndex_mean value/100). For determining the relatedness of CDR3 amino acid sequences across samples, the CalcPairwiseDistances and ClusterSamples option were used. For visualizing relatedness of CDR3 amino acid sequences across samples by phylogenetic tree, the CLCbio workbench was used (www.qiagenbioinformatics.com). For determining TCR repertoire overlap across samples, the tcR package (www.imminfo.github.io/tcr/) supported in R (www.r-project.org) was used. For visualization of this overlap, the heatmap.2 function was used^21,22^. Multiple alignment of CDR3 amino acid sequences from clones ≥33 unique UMIs from 20 samples representing 18 MS patients were also performed through CLCbio workbench to investigate clonal relatedness. The sequence WebLogos from 12 groups of related clonotypes was used to identify the amino acid motifs (www.weblogo.berkeley.edu/logo.cgi).

### Statistics

To analyze differences in the number of unique TCR-β clonotypes, diversity of TCR repertoire and clonal expansion by groups: HAM/TSP patients, MS patients and HC, we applied ANOVA followed by TukeyHSD post hoc testing under BH multiple comparison correction condition. Statistical significant differences were reached for P value < 0.05. TCR clonal size was interrogated based on the 3 groups of clones: singletons (1 unique UMI), clones ≥2 < 8 unique UMIs and clones ≥8 unique UMIs. R squared correlation coefficient (R^2^) calculation was performed to address whether the number of unique clonotypes correlates with the number of unique UMIs, as well as whether there is a correlation of the TCR clonal expansion with proviral load in HAM/TSP patients. In our longitudinal study, the 1^st^ time-point was used to investigate the clonal expansion and diversity of TCR repertoires. All graphics were generated using either R (www.r-project.org) or GraphPad Prism 6 (www.graphpad.com).

## Results

### Deep sequencing of TCR-β repertoire by UMI-filtering based analysis

**Figure 1:**
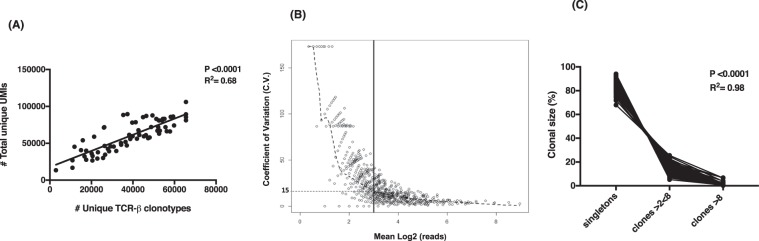
TCR sequencing analysis and clonal size of TCR repertoire in HAM/TSP patients, MS patients and HC. (**A**) Correlation of total number of unique TCR-β clonotypes and total number of unique UMIs generated by high throughput sequencing (HiSeq2500 platform, Illumina system); (**B**) Calculation of coefficient of variation (CV) using the frequency distribution of clonotypes >0 unique UMIs from the technical triplicates. Log 2^3^ of reads (more than 8) is represented by the black line, which indicates the CV = 15.0% and (**C**) Evaluation of the (%) clonal size of TCR repertoire including (1) singletons -1 unique UMI, (2) clones ≥2 < 8 and (3) clones ≥8 unique UMIs.

On average, 5.6 million sequences were generated per sample and analyzed using the MiGEC pipeline, which resulted in a high percentage ( > 90%) of TCR sequences that were removed from the initial sequencing dataset (Supplementary Section, Table 1). This reinforces the strength of the UMI-based filtering approach and correction methods in defining an accurate TCR repertoire. The number of sequences with unique UMIs was significantly and positively correlated with the number of unique TCR-β clonotypes in all subjects (P < 0.0001, R^2^ = 0.68) (Fig. 1A).

To interrogate the TCR clonal expansion, we first modeled the coefficient of variation (CV) for each clonotype by the average number of unique UMIs observed across technical triplicates (Fig. 1B). From inspection of this model (Fig. 1B), exponential variability in number of unique UMIs can be seen for those clones at an inflection point on the curve of less than 8 unique UMIs (2^3^ read count) that was associated with a CV~ 15.0% Based on this observation, we classified the TCR clones in each subject into one of 3 groups: (1) singletons or clones with 1 unique UMI; (2) clones with ≥2 < 8 unique UMIs and (3) expanded clones with ≥8 unique UMIs. As shown in Fig. 1C, for all subjects the number of clones classified as singletons was significantly greater (mean 83.8% ± 5.1) than clones with ≥2 < 8 unique UMI (mean 14.8% $\pm$ 4.2) and expanded clones ≥8 unique UMI (mean 1.2% ± 1.3) (P < 0.0001, R^2^ = 0.98). Based on these observations, we have strong confidence that designation of a clonal T-cell population in the peripheral blood of all subjects can be measured as the frequency of clones with ≥8 unique UMIs, which represents the smallest portion of the TCR repertoire (range of 0.2–6.7%).

### Higher clonal TCR expansion is inversely correlated with TCR diversity in HAM/TSP patients

**Figure 2:**
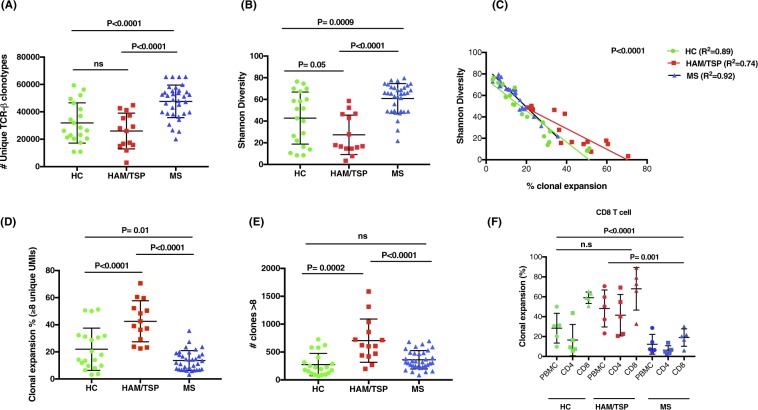
TCR clonal expansion and diversity of TCR repertoire in HAM/TSP patients, MS patients and HC. (**A**) Number of unique TCR-β clonotypes; (**B**) Diversity of the TCR repertoire; (**C**) Correlation of clonal expansion and diversity; (**D**) Clonal expansion of TCR repertoire by frequency of clones ≥8 unique UMIs and (**E**) Number of clones ≥8 unique UMIs; (**F**) Clonal expansion of TCR repertoire in PBMC, CD4^+^ and CD8^+^ T cell subsets by frequency of clones ≥8 unique UMIs (Significant differences between groups was shown for CD8 T cells). Comparison between group’s subjects were tested by ANOVA followed by TukeyHSD post hoc testing under multiple comparison in correction condition. Statistic significant differences are shown when P value was >0.05.

To determine if there were differences in the TCR clonal repertoire between HC and patients with neurological immune-mediated disorders including HAM/TSP and MS patients, we analyzed the total number of unique TCR-β clonotypes (including all 3 TCR groups, clonotypes ≥ 1 unique UMI count) from 2 million PBMCs. As shown in Fig. 2A, the number of total unique TCR-β clonotypes was significantly higher in MS patients compared to either HAM/TSP patients (P < 0.0001) or HC group (P = 0.0001). However, no significant differences were found in the number of total unique TCR-β clonotypes between HAM/TSP patients and HC (P = 0.2).

Knowing the total number of unique TCR-β clonotypes, the diversity of the TCR repertoire can be assessed by applying the Shannon Entropy algorithm^23^, which normalizes the number of unique T-cell clonotypes by the total number of sequences per subject. A significantly higher diversity within the TCR repertoire was also observed in the peripheral blood of MS patients compared to other group’s subjects (P < 0.001 for HAM/TSP and P = 0.0009 for HC) (Fig. 2B). However, the diversity of TCR repertoire in HAM/TSP patients was significantly decreased compared to HC (P = 0.05). To dismiss if these observations were due to diffrences in age, an age matched subset of MS patients were re-evaluated with HAM/TSP and HC (Supplementary Section, Fig. 1A). Results show that the higher diversity observed within the TCR repertoirte of MS patients remained significantly higher (Supplementary Section, Fig. 1B) and there was no correlation between diversity and age (Supplementary Section, Fig. 1C). Given that HAM/TSP patients had the lowest diversity of TCR repertoires compared to MS patients and HC, we hypothesized that the clonal T-cell expansion would be inversely correlated with the diversity of TCR repertoire. As shown in Fig. 2C, the increase in T cell clonal expansion significantly correlated with a decrease in the diversity of TCR repertoires in all subject’s group (P < 0.0001, R^2^ = 0.89 for HC; R^2^ = 0.74 for HAM/TSP and R^2^ = 0.92 for MS).

As mentioned, the frequency (%) of TCR clonal expansion was analyzed in all subjects by selecting clones with ≥8 unique UMIs as a percentage of the total repertoire of an individual normalized to 100%. Representative analyses from 3 HAM/TSP patients, 3 healthy controls and 3 MS patients are shown in Supplementary Section, Fig. 2. In these pie charts, singletons and clones ≥2 < 8 unique UMIs are visualized in the white area where expanded clones (≥8 unique UMIs) are shown as colored wedges. Each wedge represents a unique clonotype with a defined CDR3 sequence. While individual variations of clonally expanded T cells are observed in all groups, HAM/TSP has the highest frequency of expanded T cells compared to HC and MS patients. These results are quantitated in Fig. 2D where each dot represents the frequency of expanded T cell clones in the 3 cohorts. HAM/TSP patients showed the highest degree of TCR clonal expansion compared to either MS patients or HC (P < 0.0001). By contrast, MS patients showed the lowest TCR clonal expansion, even when compared to HC (P = 0.01) (Fig. 2D). In addition to this analysis of the frequency of TCR clonal expansion, we also asked whether there were any differences in these cohorts in the number of clones with ≥8 unique UMIs. As shown in Fig. 2E, more expanded clones were again observed in HAM/TSP patients compared to either MS patients (P < 0.0001) or HC (P = 0.0002). There were no differences in the number of clones with ≥8 unique UMIs between MS patients and HC (Fig. 2E).

**Figure 3:**
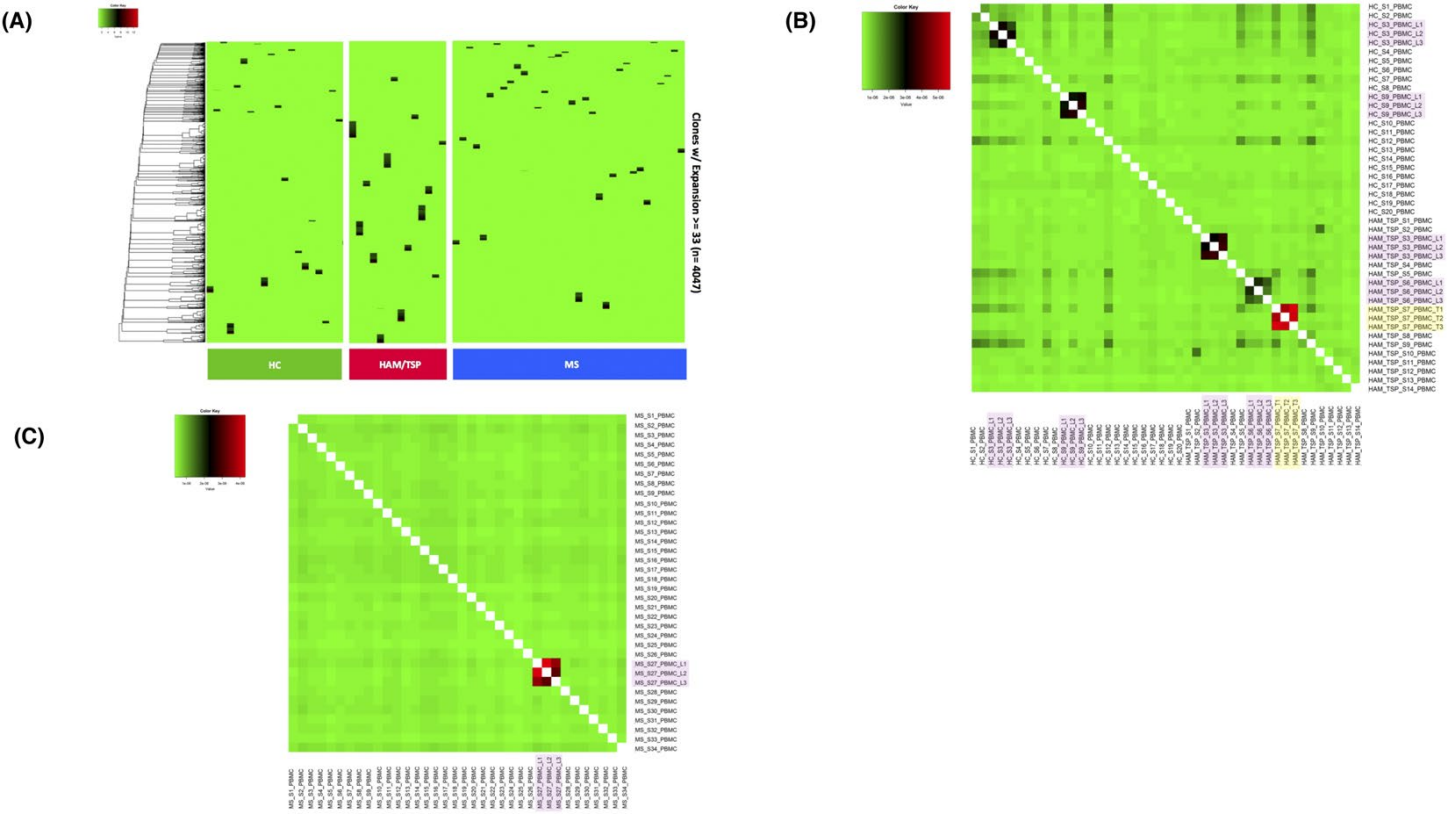
Analysis of TCR-β clonal repertoire of HAM/TSP patients, MS patients and HC by comparing the exact CDR3 amino acid sequences. (**A**) Heat map analysis represents the TCR clonal expansion (clones ≥33 unique UMIs); (**B**) Heat map analysis represents clones >0 unique UMI from HAM/TSP patients and HC, followed by three longitudinal samples from 2 HAM/TSP patients, 2 normal donors (pink highlighted samples) and a technical triplicate (yellow highlighted samples); (**C**) Heat map analysis represents clones >0 unique UMIs from MS patients, followed by two longitudinal samples (pink highlighted samples).

We also investigated the TCR clonal expansion (clones ≥ 8 unique UMIs) in purified CD4^+^ and CD8^+^ T cells subsets from 5 HC, 5 HAM/TSP patients and 5 MS patients (Fig. 2F). As expected^24^, all subjects demonstrated higher TCR clonal expansions in CD8^+^ T cells compared to CD4^+^ as graphically represented in Supplementary Section, Fig. 3 and quantitated in Fig. 2F.

### Private TCR-β repertoires in patients and healthy controls

**Figure 4:**
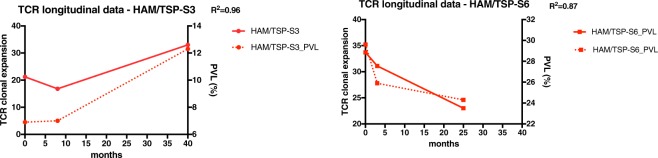
Correlation of TCR-β clonal expansion and proviral load (%) in HAM/TSP patients. This longitudinal TCR repertoire analysis was performed at baseline and followed up by 40 months in HAM/TSP-S3, or by 25 months in HAM/TSP-S6. R squared (R^2^) coefficient value is shown.

As HAM/TSP patients are associated with virus-specific immune responses restricted to immunodominant HTLV-I epitopes^25^, and MS is an inflammatory immunity-mediated disorder of unknown etiology, it was of interest to determine if there were common or “public” clonal repertoire profiles that were shared within each cohort. To test this hypothesis, we first compared the CDR3 amino acid sequences from T cell repertoires analyzed from all HAM/TSP patients, MS patients and HC. One CDR3 amino acid sequence identifies one or a small of TCR-β clonotypes recognizing the same MHC-peptide complex. We evaluated the profile of the expanded TCR repertoire using the CDR3 amino acid sequences from the most expanded clones in all subjects. To define the set of top expanded clones, we chose those clones having equal or more than 33 unique UMIs (total of 4,047 clones) since for clones with less than 33 unique UMIs across technical triplicates, the correlation on the hierarchy rank of clones was below 0.90 (Supplementary Section, Fig. 4).

Figure 3A, is a heat map analysis in which clonotype relatedness (y axis) for each individual within a cohort (x axis) can be visualized by the bin size. Two major observations can be appreciated; 1) each individual within a cohort has an expanded TCR repertoire (clones ≥33 unique UMIs), and; 2) there are no shared TCR-β clonotypes (as defined by identical CDR3 amino acid sequences) across HAM/TSP patients, MS patients and healthy control subjects.

A more detailed analysis of the TCR-β clonal repertoires between HAM/TSP patients and HC are shown in Fig. 3B, where we now evaluate the CDR3 amino acid sequences shared across HAM/TSP patients and HC cohorts by using the whole TCR-β repertoire. In this analysis, represented also as a heat map plot, similarity of the TCR-β repertoire were represented by intensity of colors, where transition from green to red describes the increase of relatedness across clonal repertoires by individual when compared to any other (Fig. 3B). Technical triplicates (HAM/TSP patient S7-T1, T2, T3) were included in this analysis along with longitudinal samples (L1, L2, L3) for: HAM/TSP patient S3, HAM/TSP patient S6, HC-S3, and HC-S9. As expected, clones representing the technical triplicates were virtually identical (Fig. 3B, yellow highlighted samples) confirming the reproducibility and robustness of this technique for sequencing of TCR-libraries. TCR-β clonal repertoires from longitudinal time-points for both HAM/TSP patients and HC were also highly related across clones (Fig. 3B, pink highlighted samples). Again, we demonstrate that there were no shared TCR-β repertoire profiles detected in healthy controls or patients with HAM/TSP. Even though all HAM/TSP patients are infected with the same virus^26^, there were no T cell clonotypes with identical CDR3 amino acid sequences that were shared among any of these HAM/TSP patients where each individual (either HAM/TSP, MS patient, or HC, Fig. 3A) has a ‘private’ TCR clonal repertoire. Moreover, longitudinal samples from the same individual had highly similar TCR-β profiles that were highly similar over time (total of 3 time-points per individual, interval from 3 to 40 months). Similarly, analyses of MS patients TCR-β repertoires, also showed a lack of any public clones that were shared among patients (Fig. 3C) where 2 longitudinal samples from one MS patient (MS-S27) again showed highly similar TCR-β profiles over time. Collectively, these results demonstrate that private TCR-β repertoires (as defined by identical CDR3 amino acid sequences) exists not only in healthy subjects, but also in patients with neurological immune-mediated diseases like HAM/TSP and MS in which TCR-β repertoire analysis can identify and track TCR signatures across individuals.

### Longitudinal TCR-β clonal expansion correlates with proviral load in HAM/TSP patients

As HAM/TSP is an inflammatory neurological disease associated with a virus infection, this afforded us the opportunity to address whether the expanded T-cell clonotypes in HAM/TSP patients (Fig. 2) correlated with the HTLV-I viral load^27^. In our cohort of 14 HAM/TSP subjects, the HTLV-I tax PVL ranged between 5.2% and 38.0% and in a cross-sectional analysis did not correlate with the percent TCR clonal expansion (Fig. 2D) (P = 0.79, data not shown). However, for 2 HAM/TSP patients (HAM/TSP-S3 and -S6) that have multiple samples spanning 40 months and 25 months, respectively, TCR-β clonal expansion did correlate with the levels of HTLV-I proviral load (Fig. 4). While HAM/TSP-S3 showed an increase in clonal T-cell expansion associated with increased HTLV-I PVL (R^2^ = 0.96), there was an inverse correlation in HAM/TSP-S6 that demonstrated decreased clonal T-cell expansion associated with a decrease in PVL (R^2^ = 0.87). These results suggest that the clonal T-cell expansion in the peripheral blood of HAM/TSP patients reflect the immune response against HTLV-I infection.

### Dissecting a TCR-β repertoire signature in MS

**Figure 5:**
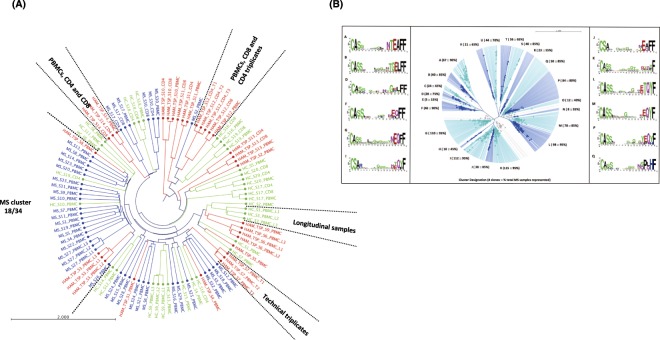
Phylogenetic tree analysis representing the similarities on the CDR3 amino acid sequences of TCR repertoire. (**A**) Analysis of TCR repertoire was performed in PBMCs, CD4^+^ and CD8^+^ T cells subsets from 14 HAM/TSP patients (coded in red color), 34 MS patients (coded in blue color) and 20 HC (coded in green color); (**B**) Analysis of the TCR clonal relatedness was performed in PBMCs from MS patients, ‘MS cluster’ (n = 18). In (**A**) clonotypes were selected by using the *VDJtools*, through CalcPairwiseDistances analysis and clustered data was imported into the CLCbio run in order to generate a representative phylogenetic tree. A total of 106 TCR-libraries were incorporated in this analysis including longitudinal samples (L1, L2, and L3) and technical triplicates (T1, T2 and T3). In (**B**) clonotypes with ≥33 unique UMIs were selected and relatedness of TCR clonal expansion was also investigated using CLCbio workstation to generate a representative phylogentic tree. A total of 12 groups of related clones were selected and amino acid sequence motif was represented as WebLogo.

Since non-shared or “private” TCR clonal repertoires were observed across all cohorts, HAM/TSP, MS and HC, when we compared TCR clonotypes using a stringent ‘identical’ CDR3 amino acid sequences approach (clones ≥33 unique UMIs) (Fig. 3B,C), we employed a less stringent phylogenetic tree analysis using clonotypes with at least one unique UMI in conjunction with diversity metrics to distinguish groups (Fig. 5A). This approach performs an all-versus-all pairwise comparison of clonotypes detected for a list of samples and computes a set of repertoire similarity measures based on CDR3 amino acid sequence; including the pearson correlation of clonotype frequencies, the diversity of overlap, and the geometric mean for the overlap. These metrics are then used to hierarchical cluster samples, producing a sample-level dendrogram. Upon visualization of this dendrogram, branch lengths describe the distance between repertoires (longer the branch length = greater dissimilarity per repertoire relatedness by CDR3 amino acid sequence and greater discordance per observed clonotype frequency)^28^.

To demonstrate the accuracy and robustness of our TCR repertoire UMI-filtering based approach, we included 106 TCR-β repertoires from 20 HC, 14 HAM/TSP and 34 MS patients, technical replicates (HAM/TSP = 3), longitudinal samples (HAM/TSP = 4; HC = 4; MS = 2) and pairs of sorted CD4^+^ and CD8^+^ T-cell subsets from HC (N = 10), HAM/TSP patients (N = 11), and MS patients (N = 4).

In Fig. 5A, the similarities in the TCR-β repertoires are shown in green for the HC, red for HAM/TSP, and blue for MS patients. As expected, technical replicates and longitudinal samples from the same patient grouped together (Fig. 5A, examples noted). Likewise, CD4^+^ and CD8^+^ TCR samples also grouped with their respective PBMCs. Again, these results demonstrated the sensitivity and accuracy of our technical strategy to study the TCR repertoires by HTS. Importantly, there was no exclusive TCR-β repertoire clusters observed for HC (green) or HAM/TSP patients (red). However, a subset of 20 MS samples representing 18/34 MS patients appeared to group (Fig. 5A, “MS cluster”). This analysis suggests the existence of a TCR-β repertoire-level signature in at least half of the MS cohort. There was no correlation between any demographic, clinical or radiological outcomes including age, gender, ethnicity, number of relapses, expanded disability status scale (EDSS) score and number of MRI contrast enhanced gadolinium positive lesions (CEL) by MRI for those patients grouped within the “MS cluster” versus MS patients not grouping with this cluster (Supplementary Section, Table 3). Also, no differences in the numbers of unique TCR-β clonotypes, percent clonal expansion or diversity of TCR repertoires were found (data not shown).

Since MS patients have a ‘private’ or non-shared TCR repertoire, we further investigated whether there was any related CDR3 amino acid sequence across 20 samples representing the 18 MS patients definied within the ‘MS cluster’. Relatedness of the top expanded clones (≥33 unique UMIs) was evaluated by using phylogenetic tree analysis (total of 1,128 clones). As shown in Fig. 5B, we identified 22 groups of related clonotypes across these MS patients. For each of these groups (A-V), the number of clones and percentage of total MS patients is described. For example, in group A, 67 clones were observed to be related by CDR3 amino acid sequence in 18 samples (90%). Group details for all clones are shown in Supplementary Section, Table 4, while the consensus CDR3 amino acid sequence observed for groups with a percentage of total MS patients greater than 75% (12 groups) is represented as a Web Logo (Fig. 5B). Presence of the amino acid glicine (G) at positions 9 to 12 was seen in some sequences motif (B, F, G, I, L, M, P and Q).

## Discussion

Analyzing TCR repertoires may help to gain a better understanding of immune-mediated responses in neuroinflammatory diseases, in particular in those disorders of unknown etiology, such as MS, but in which virus(es) have long been suspected as antigenic triggers^29^. MS is a chronic neuroinflammatory disease in which peripheral immune cells are thought to cross the blood-brain barrier (BBB) into the CNS and initiate a cascade of events that are deleterious to resident glial and neuronal cells. Identification of TCR clonotypes and their CDR3 amino acid sequences may therefore help to better characterize immune responses in MS and may give insights into the antigen or ‘triggers’ of these responses. As a comparative group, we have also analyzed TCR repertoires of patients with a chronic, progressive myelopathy, clinically similar to some forms of MS, but known to be associated with an exogeneous human virus, HTLV-I^30^. Patients with HAM/TSP are characterized by activated T cells in both the periphery and CSF in which virus-specific T-cells play an important role in the pathogenesis of this disease^31^.

Previous studies in MS have applied HTS of TCR-β chain repertoires to the investigation of brain-infiltrating T cells in demyelinating lesions from autopsy tissue and have identified shared clones across peripheral blood and CNS^12^. Sequencing of TCR repertoires has also been used to assess the clonal expansion and frequency of EBV-reactive T-cell clonotypes shared between the peripheral blood and CSF in MS patients^32,33^. Previously, we also analyzed the TCR repertoire in paired peripheral blood and CSF T-cells of MS patients. While we observed that the most frequent clonotypes were predominantly unique to each compartment, there were clonally expanded TCR repertoires in MS patients in comparison to a non-neurological disease control group^13^. Due to a variety of technical limitations^34^, accurately measuring TCR clonal expansion has been a challenge in this field. Here we have applied a more comprehensive and robust TCR sequencing methodology to accurately evaluate TCR clonal expansion that combines the 5′RACE amplification approach with unique molecular identifier (UMI) bar codes and a post-UMI filtering based MiGEC process to track each individual cDNA molecule while correcting any artificial CDR3 amino acid sequence^20^. In support of this analysis, technical triplicates were added to precisely calculate the variability in frequency of each individual clone.

As HAM/TSP is a virus associated immunopathologically mediated disease, it was not surprising that in PBMC there was a higher TCR clonal expansion compared to HC and patients with MS. Consistent with previous reports^35^, there was a significantly higher clonal expansion in CD8^+^ T-cell subset compared to CD4^+^ T-cells in all cohorts tested. With a higher TCR clonal expansion it was expected that this would coincide with a decrease in TCR repertoire diversity which indeed was observed in patients with HAM/TSP. Surprisingly, MS patients had an increase in TCR diversity (and decreased TCR clonal expansion) even greater than in HC. While this could be related to age differences between cohorts, this is unlikely since age-matched subsets of MS patients, HAM/TSP, and HC continued to demonstrate significantly higher TCR repertoire diversity in MS patients. In HC, it has been reported that increased TCR diversity is also correlated with an increase in the percentage of naïve CD45RA^high^/CD27^high^ T-cells^20^. It has been postulated that post thymic proliferation of central naïve CD4^+^ T cells can be driven by self-peptides, resulting in a selection and expansion of potentially autoreactive T_helper_ cells. Increase of autoimmune naïve CD4^+^ T cells has been suggested^36^ to facilitate the initiation of autoimmune diseases in susceptible individuals. Indeed, thymic dysfunction resulting in increased T-cell turnover has been associated with autoimmune disease such as rheumatoid arthritis and multiple sclerosis^37,38^. In the present MS cohort which was shown to have increase TCR diversity (Fig. 2B), there was also a concomitant increase in naïve CD45RA^high^/CD27^high^ T-cells compared to HC or patients with HAM/TSP (data not shown).

As we have demonstrated an increased TCR clonal expansion in patients with HAM/TSP, we postulated that these expanded T cell clonotypes would be driven by the HTLV-I proviral DNA detected in peripheral blood. A TCR repertoire longitudinal study was performed that included 3 longitudinal time-points from two HAM/TSP patients, one in which the viral load increased over 40 months while the other patient had a decrease in viral load over 2 years. In both patients, the levels of HTLV-I proviral DNA correlated with the levels of TCR clonal expansion supporting the hypothesis that the amount of virus in the peripheral blood drives TCR clonal expansion. Since all the T cell clonotype VDJ CDR3 β-chain amino acid sequences are known within these expanded TCR repertoires it remains to be determined what antigenic specificities these T cell clones recognize. It is compelling to consider that expanded T cell clonotypes from HAM/TSP patients may be specific for HTLV-I proteins such as HTLV-I tax, since it is known that in HLA-A*0201 HAM/TSP patients, the frequency of Tax specific CD8^+^ T-cells is extraordinarily high in peripheral blood and even higher in CSF^39,40^. Although beyond the scope of this report, the ability to decode antigen specificities contained within the sequences of the rearranged TCR is an area of intense investigation^21^ and studies of TCR clonal repertoires in viral associated diseases such as HAM/TSP will be of use in these endeavors^11^.

It is expected that in a viral-triggered, neuroinflammatory disease such as HAM/TSP in which antigen-specific T cells are thought to be immunopathogenic, expansion of TCR repertoires would be observed. It is less clear, if expanded TCR repertoires would be seen in PBMC of patients with MS, although it has been reported^13^. Using a highly conservative approach to define an expanded T cell clonotype (clones with greater than 33 unique UMIs), heat map analysis based on CDR3 amino acid sequences demonstrated individual or “private” TCR-β signatures in all cohorts. No expanded (‘public’) clonotype was observed within any cohort (even HAM/TSP patients) or across groups. We extended this analysis using total T-cell clonotype repertoires including technical triplicates and longitudinal specimens obtained from HAM/TSP and HC. Our findings demonstrated that (i) the immune TCR profile did not significantly change overtime, where an individual’s TCR signature can be identified in a longitudinal study; (ii) a high conservative rank of clonotypes were maintained in the technical triplicate analysis and again, (iii) very few shared clonotypes were observed among the subjects based on the CDR3 amino acid sequences. Since these results indicated that a private TCR repertoire exists among HAM/TSP patients, MS patients and HC based on comparisons of identical CDR3 sequences, we used a less conservative phylogenetic tree analysis to explore if any TCR repertoire relatedness using clonotypes with at least one unique UMI in conjunction with diversity metrics (Fig. 5). While there were no exclusive TCR-β repertoire clusters observed for HC (green) or HAM/TSP patients (red), we identified a cluster of highly related T cell clonotypes observed in a subgroup of MS patients (18 out of 34). This suggests that a common TCR repertoire-level ‘signature’ might characterize a group of MS patients and distinguish them from HC and at least one other group of patients with a neurologically immune-mediated disease such as HAM/TSP. It remains to be determined if this subgroup of MS patients defined by this cluster of TCRs will have a different disease course, clinical prognosis, or response to treatment. While there was no apparent clinical or radiological correlate associated with this subset of patients, clearly larger cohorts of MS patients will be needed to assess the significance of this TCR cluster signature. Alternatively, since each patient has their own ‘private’ TCR signature, it will be of interest to determine if this group of T cell clonotypes again changes with time or treatment.

While we have identified a TCR repertoire-level ‘signature’ that might characterize a group of MS patients, it remained to be determined if there were common CDR3 motifs that may define this cluster. We addressed this by using clones ≥33 unique UMIs (correlation of ranks 0.9, Supplementary Section, Fig. 4), which represents the most expanded clones of the TCR repertoire. Phylogenetic tree analysis revealed 22 groups of related clones each having a separate consensus CDR3 amino acid motif. A conservation of the amino acid glycine (G) was observed at postion 9–10 across these motifs (Fig. 5B) similar to a previous report^13^. Even though each patient has thier own ‘private’ TCR repertoire, a relatedness of clones may exist across the TCR repertoire. Larger cohorts of patients with other neurologic and immune mediated disorders will need to be evaluated to determine the disease specificity of the CDR3 amino acid motif.

In summary, we demonstrated that deep sequencing of cDNA TCR-β repertoire by using a UMI-filtering based analysis generated a more comprehensive and robust dataset, allowing us to evaluate with confidence the TCR clonal expansion in patients with HAM/TSP or MS. Here we reported a relatedness of clonotypes in a group of MS patients, suggesting that a TCR signature might characterize these patients.

## Electronic supplementary material

Supplementary Section
