# Supplementary material for: Comprehensive Analysis of TCR-β Repertoire in Patients with Neurological Immune-mediated Disorders
Source: Sci Rep. 2019 Jan 23;9:344. doi: 10.1038/s41598-018-36274-7 (PMC6344574; doi:10.1038/s41598-018-36274-7)

Comprehensive Analysis of TCR-β Repertoire in Patients with Neurological Immune-mediated Disorders

Alessandra de Paula Alves Sousa[1], Kory R. Johnson[2], Joan Ohayon[3], Jun Zhu[4], Paolo A. Muraro[5] and Steven Jacobson*[1]

1. Neuroimmunology Branch, Viral Immunology Section, National Institute of Neurological Disorder and Stroke, NIH, Bethesda, Maryland.
2. Bioinformatics Section, National Institute of Neurological Disorder and Stroke, NIH, Bethesda, Maryland.
3. Clinical Neuroimmunology Unit, National Institute of Neurological Disorder and Stroke, NIH, Bethesda, Maryland.
4. Systems Biology Center, National Heart Lung and Blood Institute, NIH, Bethesda, Maryland.
5. Division of Brain Sciences, Faculty of Medicine, Imperial College London, London, United Kingdom.

*Correspondent author:

Steven Jacobson
Neuroimmunology Branch, Viral Immunology Section, National Institute of Neurological Disorders and Stroke, 9000 Rockville Pike, Clinical Research Center, Room 5C100, Bethesda, Maryland, USA
Phone: +1 301 496 0519
FAX: +1 301 402 0373
E-mail address: jacobsons@ninds.nih.gov

Conflict of interest statement:
The authors have declared that no conflicts of interest exist.

Supplementary Section

Table 1- Demographic information and TCR-β dataset statistics from HAM/TSP patients, MS patients and healthy controls.

Subject	Sex	Age	Ethnicity	# Total HTS sequences	# Total unique UMIs	# Unique TCR-β clonotypes	% Clonal expansion	Shannon Diversity
HC-S1	M	75	African American	9412373	58981	21321	50.6	8.4
HC-S2	F	66	Caucasian	5408124	29258	22876	14.0	51.3
#HC-S3_L1	F	46	African American	6278303	47117	37035	6.0	71.8
#HC-S3_L2	F	47	African American	5454027	31015	23479	7.4	66.2
#HC-S3_L3	F	48	African American	4112939	53035	40978	6.0	71.8
HC-S4	F	72	Caucasian	8609894	71871	26193	51.3	10.5
HC-S5	M	67	Caucasian	6874922	32872	20559	17.8	42.5
HC-S6	M	43	African American	6598607	86250	59371	14.2	67.3
HC-S7	F	43	African American	3423784	39231	28518	9.7	58.6
HC-S8	F	59	Caucasian	4948655	39535	31976	3.2	74.6
#HC-S9_L1	M	33	Caucasian	5887410	87750	49410	28.2	34.8
#HC-S9_L2	M	34	Caucasian	6506710	85146	44200	35.2	25.1
#HC-S9_L3	M	35	Caucasian	6131015	59875	31487	33.5	26.5
HC-S10	M	60	Caucasian	5689963	56237	41930	8.2	70.0
HC-S11	M	68	Caucasian	5960212	48057	34345	11.9	60.1
HC-S12	F	66	Caucasian	5625460	27676	17469	28.8	21.9
HC-S13	M	57	Caucasian	6346628	66319	56311	3.7	76.5
HC-S14	F	57	Caucasian	7674115	71905	52154	13.4	51.7
HC-S15	F	44	African American	8801824	87085	46406	31.8	26.4
HC-S16	M	59	Caucasian	10620981	16813	10870	30.6	13.8
HC-S17	F	43	Caucasian	11058000	26959	10846	50.1	8.5
HC-S18	M	29	Caucasian	9374668	46432	26335	31.3	16.4

HC-S19	F	49	Caucasian	9335805	26737	20456	9.8	58.4
HC-S20	F	56	African American	10543066	37837	23137	20.4	40.0
HAM/TSP-S1	M	70	African American	6755209	89501	37368	39.1	42.9
HAM/TSP-S2	M	62	Caucasian	7142277	45303	11824	60.5	14.6
#HAM/TSP-S3_L1	M	39	Caucasian	6398450	67652	42625	21.3	48.6
#HAM/TSP-S3_L2	M	40	Caucasian	7023683	51931	36049	16.9	54.0
#HAM/TSP-S3_L3	M	42	Caucasian	7524752	66014	33387	33.2	36.6
HAM/TSP-S4	F	68	Caucasian	7387766	78549	41294	36.3	27.9
HAM/TSP-S5	F	59	Caucasian	7567321	71372	26038	52.2	7.6
#HAM/TSP-S6_L1	F	49	African American	7595479	85477	44852	33.9	46.5
#HAM/TSP-S6_L2	F	49	African American	6186521	105712	58857	31.2	50.1
#HAM/TSP-S6_L3	F	51	African American	5288227	100915	65878	22.9	60.3
&HAM/TSP-S7	M	66	Caucasian	6834643	45758	29226	22.5	50.2
HAM/TSP-S8	F	49	African American	5753567	88395	35356	48.4	14.7
HAM/TSP-S9	F	49	African American	11295085	39904	17509	42.6	16.5
HAM/TSP-S10	M	59	African American	11890855	13421	2923	70.6	3.4
HAM/TSP-S11	F	62	African American	17495853	54097	15866	59.7	17.8
HAM/TSP-S12	F	24	Caucasian	15391351	40728	14982	50.4	16.8
HAM/TSP-S13	F	60	Caucasian	11945452	32215	16671	37.2	15.7
HAM/TSP-S14	F	38	African American	14722795	42478	27936	23.2	46.8
MS-S1	M	22	African American	2365398	81221	54074	17.9	48.9
MS-S2	F	36	African American	1868059	36085	20110	23.7	40.1
MS-S3	M	36	African American	1704866	67094	51819	6.6	74.6
MS-S4	M	44	Caucasian	2070700	59687	39852	20.0	49.1
MS-S5	M	35	African American	1356392	83140	55192	19.7	48.8
MS-S6	M	53	Caucasian	1793103	61258	43087	14.3	61.4
MS-S7	M	30	Caucasian	1689694	71952	55662	6.7	74.7

MS-S8	F	39	Caucasian	1868175	61089	46224	9.9	66.2
MS-S9	F	37	Caucasian	1555111	59494	45275	11.0	65.5
MS-S10	F	50	Caucasian	1941200	80974	52533	14.6	65.8
*MS-S11	F	50	African American	1436743	72102	48774	14.7	62.6
MS-S12	M	32	Caucasian	1933220	50378	34255	12.0	65.2
*MS-S13	F	68	Caucasian	1011521	63056	52601	6.7	68.8
MS-S14	M	50	Caucasian	2149468	67060	52977	7.7	70.8
MS-S15	F	52	Caucasian	1894461	34621	26590	7.1	68.5
MS-S16	M	51	Caucasian	1978589	51913	36443	11.2	66.1
MS-S17	F	26	Caucasian	1197939	30033	25601	6.7	68.7
MS-S18	M	43	Caucasian	5016196	81014	53850	14.3	62.9
MS-S19	F	28	Caucasian	3546348	106010	65536	23.4	48.6
*MS-S20	M	54	Caucasian	3925534	83006	54432	13.4	65.4
MS-S21	F	56	Caucasian	4802530	85438	65536	8.6	70.8
MS-S22	F	60	Caucasian	4287250	83091	51259	21.8	49.2
MS-S23	F	51	Caucasian	5104501	78074	39681	35.6	21.8
MS-S24	F	52	Caucasian	5502180	85847	58201	16.8	55.7
MS-S25	F	37	Caucasian	4704177	89321	65536	7.5	77.3
MS-S26	F	36	African American	3935097	56894	47329	7.3	71.4
#MS-S27_L1	F	63	Caucasian	4109067	45584	30744	16.6	46.8
#MS-S27_L2	F	64	Caucasian	5195268	72876	49816	10.4	45.7
#MS-S27_L3	F	65	Caucasian	5725484	122861	61429	29.5	55.9
MS-S28	M	62	Caucasian	4457452	56427	38787	19.1	47.3
MS-S29	M	36	Caucasian	3890539	61441	38189	27.4	31.5
MS-S30	F	45	Caucasian	2967997	66098	57846	3.2	73.0
MS-S31	F	70	Caucasian	1607145	74232	59797	6.4	74.0
MS-S32	F	37	Caucasian	4282869	57904	48038	6.2	79.7

MS-S33	F	30	Caucasian	4610465	81162	65536	5.9	77.7
MS-S34	F	38	Caucasian	3743273	55777	38687	18.3	47.7

*MS-S11, *MS-S13 and *MS-S20 were clinically diagnosed as secondary progressive MS, all other MS patients were relapsing remitting MS according to McDonalds criteria [19]. &TCR-β dataset statistics from HAM/TSP-S7 is the average of 3 identical samples tested (technical triplicates), and subjects HC-S3, HC-S9, HAM/TSP-S3, HAM/TSP-S6 and MS-S27 are representated by all three longitudinal time-points; HTS, high throughput sequencing; UMIs, unique molecular identifiers.

Table 2- Sequence primers used for TCR-β library construction.

**cDNA synthesis**	
**Switch-template 5'RACE with UMI**	**5'-AAGCAGTGGTATCAACGCAGAGTACNNNNNNNNNNUCTTrGrGrGrG-3'**
**Oligo dT**	**5'-TTTTTTTTTTTTTTTTTTTTTTTTTTTV-3'**
**1st PCR amplification**	
**P5**	**5-AATGATACGGCGACCACCGAGATCTACACAAGCAGTGGTATCAACGCAGAGTAC-3'**
**P7_Index_1**	**5'-CAAGCAGAAGACGGCATACGAGATAACGGAGTGCTTCTGATGGCTCAAACACAGCGACCT-3'**
**P7_Index_2**	**5'-CAAGCAGAAGACGGCATACGAGATAAGCGGTTGCTTCTGATGGCTCAAACACAGCGACCT-3'**
**P7_Index_3**	**5'-CAAGCAGAAGACGGCATACGAGATAATCCGGTGCTTCTGATGGCTCAAACACAGCGACCT-3'**
**P7_Index_4**	**5'-CAAGCAGAAGACGGCATACGAGATACAGCTGTGCTTCTGATGGCTCAAACACAGCGACCT-3'**
**P7_Index_5**	**5'-CAAGCAGAAGACGGCATACGAGATACCACGTTGCTTCTGATGGCTCAAACACAGCGACCT-3'**
**P7_Index_6**	**5'-CAAGCAGAAGACGGCATACGAGATACGAGAGTGCTTCTGATGGCTCAAACACAGCGACCT-3'**
**P7_Index_7**	**5'-CAAGCAGAAGACGGCATACGAGATACTACGGTGCTTCTGATGGCTCAAACACAGCGACCT-3'**
**P7_Index_8**	**5'-CAAGCAGAAGACGGCATACGAGATAGACAGCTGCTTCTGATGGCTCAAACACAGCGACCT-3'**
**P7_Index_9**	**5'-CAAGCAGAAGACGGCATACGAGATAGCACCTTGCTTCTGATGGCTCAAACACAGCGACCT-3'**
**P7_Index_10**	**5'-CAAGCAGAAGACGGCATACGAGATAGGTAGCTGCTTCTGATGGCTCAAACACAGCGACCT-3'**
**P7_Index_11**	**5'-CAAGCAGAAGACGGCATACGAGATAGTGGCTTGCTTCTGATGGCTCAAACACAGCGACCT-3'**
**P7_Index_12**	**5'-CAAGCAGAAGACGGCATACGAGATATCCACGTGCTTCTGATGGCTCAAACACAGCGACCT-3'**
**P7_Index_13**	**5'-CAAGCAGAAGACGGCATACGAGATATGCCAGTGCTTCTGATGGCTCAAACACAGCGACCT-3'**
**P7_Index_14**	**5'-CAAGCAGAAGACGGCATACGAGATCAACAGGTGCTTCTGATGGCTCAAACACAGCGACCT-3'**
**P7_Index_15**	**5'-CAAGCAGAAGACGGCATACGAGATCACATGCTGCTTCTGATGGCTCAAACACAGCGACCT-3'**
**P7_Index_16**	**5'-CAAGCAGAAGACGGCATACGAGATCAGACAGTGCTTCTGATGGCTCAAACACAGCGACCT-3'**
**P7_Index_17**	**5'-CAAGCAGAAGACGGCATACGAGATCCAATCGTGCTTCTGATGGCTCAAACACAGCGACCT-3'**
**2nd PCR amplification**	
**P5**	**5'-AATGATACGGCGACCACCGAGATCTACAC-3'**
**P7**	**5'-CAAGCAGAAGACGGCATACGAGAT-3'**
**Sequencing primers**	
**R1**	**5'-CAAGCAGTGGTATCAACGCAGAGTAC-3'**
**R2**	**5'-TGCTTCTGATGGCTCAAACACAGCGACCT-3'**
**Index**	**5'-AGGTCGCTGTGTTTGAGCCATCAGAAGCA-3'**

Table 3- Clinical information of MS patients who were divided in two subgroups "cluster and non-cluster", according to the TCR-β repertoire similarity by phylogenetic tree analysis. Expanded Disability Status Score , number of relapses and enhancement gadolinium positive lesion are represented as average value.

MS subgroup	Age	Gender	EDSS score	Number of relapses	Number of CEL
Cluster	45.4 (22 - 70)	12F / 6M	2 (0 - 6.5)	2.5 (1 - 5)	0.16 (0 - 4)
Non-cluster	40.9 (26 - 62)	9F / 7M	2 (0 - 6.0)	2.7 (1- 7)	1.18 (0 - 5)

EDSS: Expanded Disability Status Scale; CEL: enhancement gadolinium positive lesion; average value is shown (minimum and maximum value).

Table 4- Groups of relared clones (A - V) across 20 PBMC samples represented by 18 MS patients ('MS cluster').

SAMPLES	A	B	C	D	E	F	G	H	I	J	K	L	M	N	O	P	Q	R	S	T	U	V
**MS-S1**	2	0	0	0	0	3	10	1	7	1	6	5	3	0	1	7	2	0	0	5	0	1
**MS-S4**	4	2	1	0	0	0	6	1	5	1	4	11	6	0	0	5	3	1	3	0	4	1
**MS-S5**	5	2	0	3	0	4	7	1	11	4	9	10	2	0	0	2	1	2	0	2	3	1
**MS-S7**	0	1	0	0	0	1	2	0	1	0	4	2	2	1	0	2	1	1	0	0	0	0
**MS-S8**	1	1	1	1	0	0	3	0	3	1	6	5	4	0	0	4	2	0	0	0	0	2
**MS-S9**	2	4	1	1	0	1	3	0	1	1	5	2	3	1	0	2	1	0	1	0	3	1
**MS-S10**	7	2	3	2	0	3	11	1	5	2	11	5	0	0	1	1	4	1	0	0	0	2
**MS-S11**	2	1	1	0	1	2	6	2	3	2	3	6	7	1	0	8	1	1	2	3	1	0
**MS-S13**	2	0	2	2	2	1	0	1	1	0	2	3	1	0	0	0	0	0	0	0	0	0
**MS-S14**	0	0	0	1	0	3	5	0	3	1	3	4	4	0	0	1	1	1	1	0	1	0
**MS-S19**	2	7	4	1	0	6	14	0	12	2	13	6	12	0	0	9	8	4	4	5	10	1
**MS-S20**	5	3	3	2	0	3	3	0	1	1	3	2	3	1	0	3	1	0	4	1	1	0
**MS-S22**	10	2	3	2	2	3	14	0	3	1	12	5	1	1	1	7	2	2	3	4	3	2
**MS-S23**	2	3	2	2	0	3	8	0	8	5	9	11	8	2	1	10	5	0	7	1	3	1
**MS-S26**	2	2	1	1	0	1	2	0	0	1	1	1	2	0	1	0	0	0	0	1	0	0
***MS-S27_L1**	2	1	0	1	0	2	1	0	3	2	3	1	3	0	2	2	2	1	1	1	2	0
***MS-S27_L2**	11	4	1	3	0	2	7	1	19	7	13	9	11	0	2	13	7	6	8	7	8	4
***MS-S27_L3**	5	1	0	4	0	2	6	0	20	4	7	6	6	1	3	8	7	3	4	4	3	3
**MS-S31**	2	2	0	0	0	1	1	1	4	0	1	0	0	0	0	0	0	0	1	1	1	1
**MS-S33**	1	2	1	2	0	5	1	1	2	2	0	4	0	0	0	0	2	0	1	1	1	1

**# SAMPLES**	**18**	**17**	**13**	**15**	**3**	**18**	**19**	**9**	**19**	**17**	**19**	**19**	**17**	**7**	**8**	**16**	**17**	**11**	**13**	**13**	**14**	**13**
**# CLONES**	**67**	**40**	**24**	**28**	**5**	**46**	**110**	**10**	**112**	**38**	**115**	**98**	**78**	**8**	**12**	**84**	**50**	**23**	**40**	**36**	**44**	**21**
**% SAMPLES**	**90**	**85**	**65**	**75**	**15**	**90**	**95**	**45**	**95**	**85**	**95**	**95**	**85**	**35**	**40**	**80**	**85**	**55**	**65**	**65**	**70**	**65**

*MS-S27 is represented by three longitudinal time-points.

Figure 1 – **(A)** Statistic comparison of similarly aged MS patients (n=20), HAM/TSP (n=14), and HC (n=20); **(B)** and analysis of their diversity of TCR repertoire.

(C) Correlation of diversity of TCR repertoire with age was evaluated in all 68 subjects included in this study. Statistic significant differences are shown when P value was > 0.05.

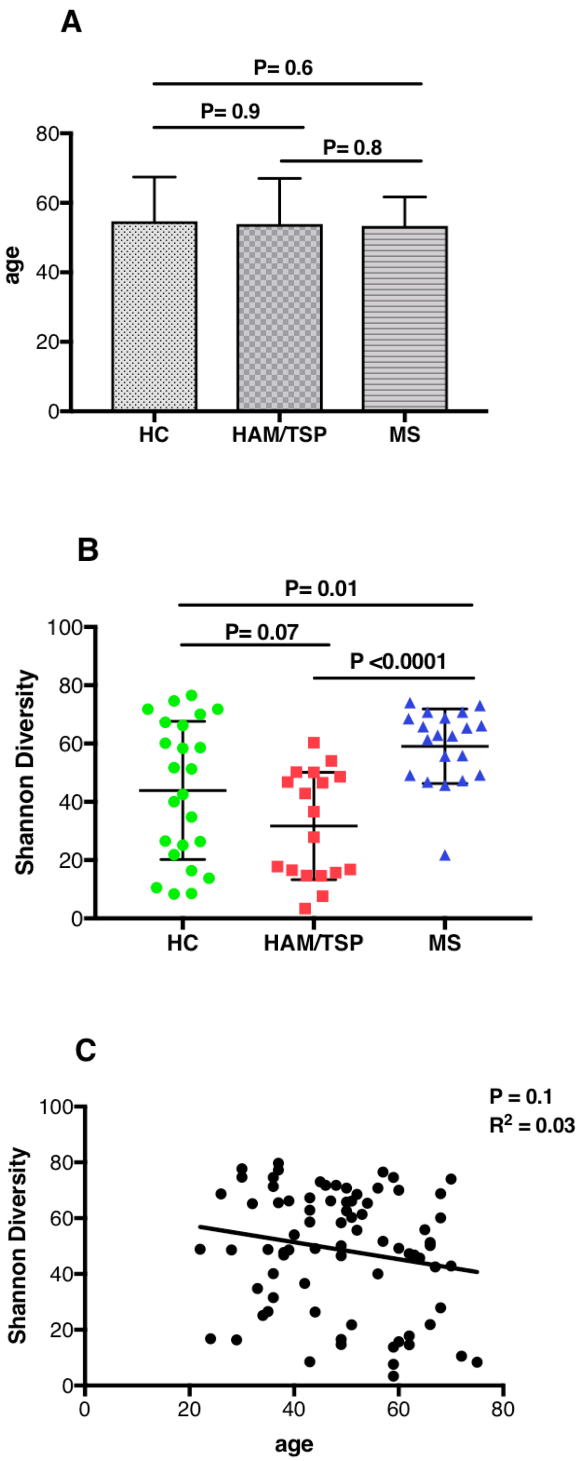

Figure 2 – Analysis of T-cell clonal expansion in PBMCs of HAM/TSP patients, MS patients and HC. TCR clonal expansion was analyzed by using the frequency of clones ≥8 unique UMIs (only three subjects per each group are shown), grey color represents both clones ≥2<8 unique UMIs and singletons (clones with 1 unique UMI).

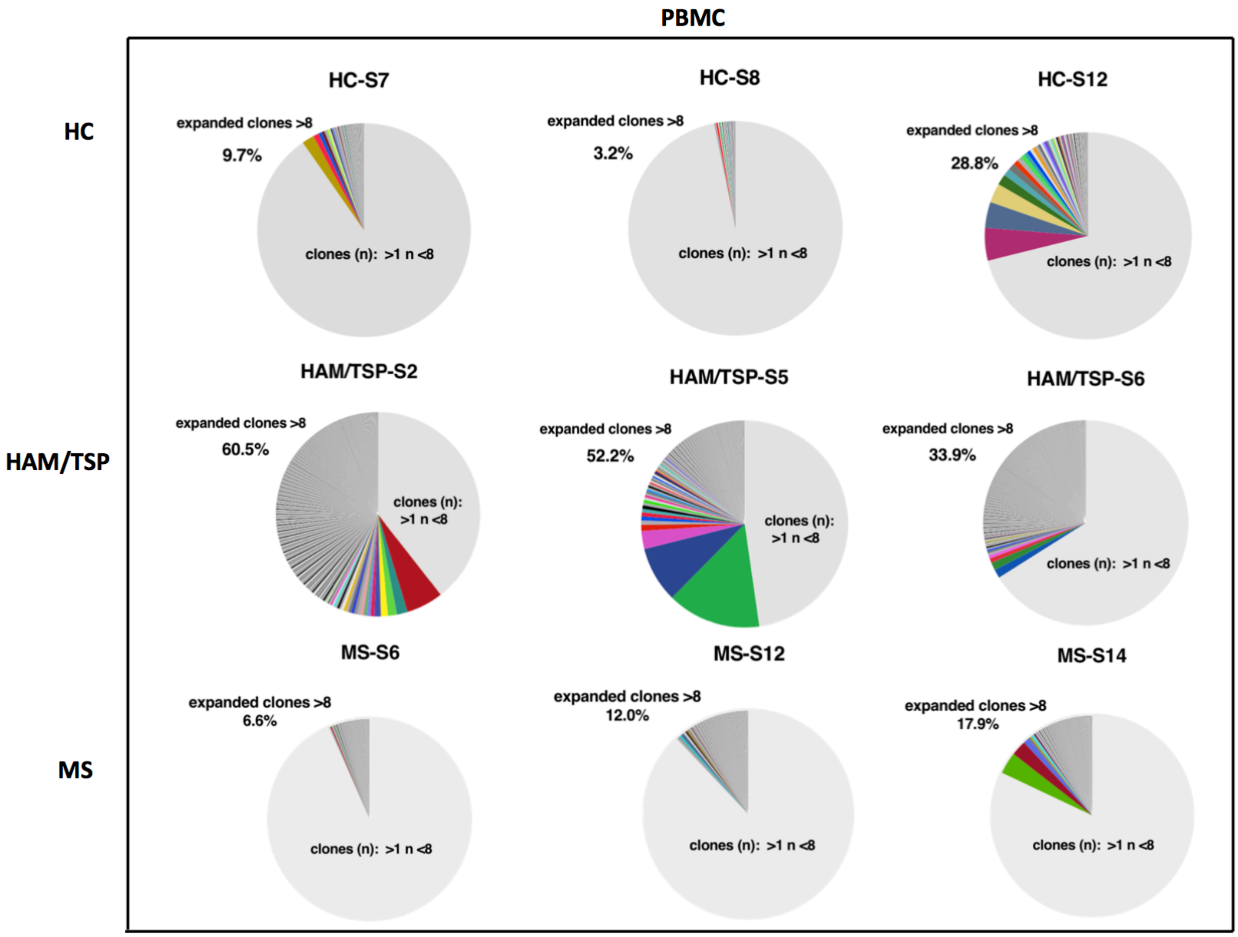

Figure 3 – Analysis of TCR clonal expansion in CD4+ and CD8+ T-cells of HAM/TSP patients, MS patients and HC. Representation of clonal TCR expansion was analyzed by using the frequency of clones ≥8 unique UMIs (only two subjects per each group are shown), grey color represents both clones ≥2<8 unique UMIs and singletons (clones with 1 unique UMI).

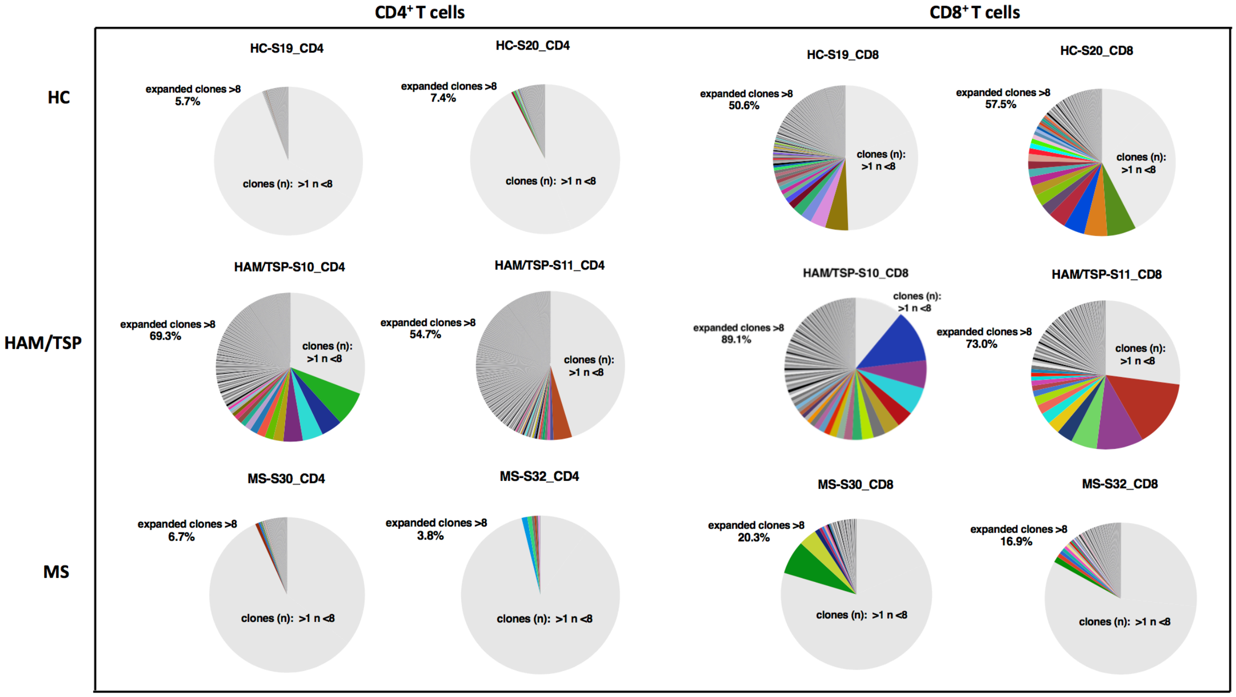

Figure 4 – Hierarchy rank of clonal T-cell repertoires based on the CDR3 amino acid sequences of three technical replicates. A correlation value of 0.9 was observed for clones ≥33 unique UMIs. Standard deviation representing the variation on the hierarchy rank of clones from technical triplicates (samples 1, 2 and 3) is shown by the grey line.

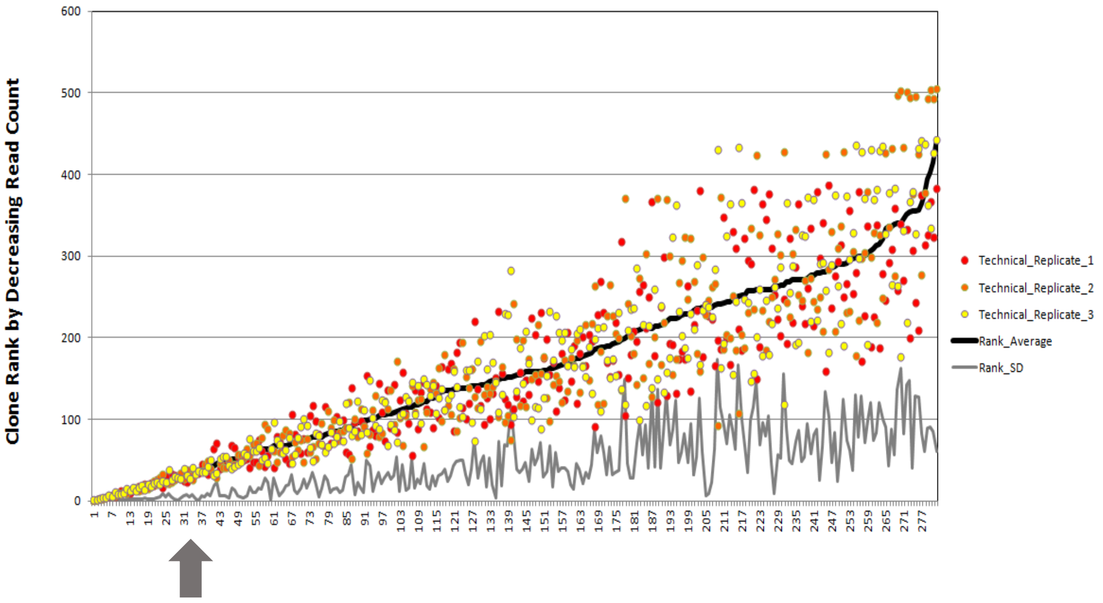

Supplement: Supplementary file 1 — Supplementary Section [file 41598_2018_36274_MOESM1_ESM.pdf]
